# Recovering the quantum formalism from physically realist axioms

**DOI:** 10.1038/srep43365

**Published:** 2017-03-03

**Authors:** Alexia Auffèves, Philippe Grangier

**Affiliations:** 1 Institut Néel, BP 166, 25 rue des Martyrs, F38042 Grenoble Cedex 9, France; 2 Laboratoire Charles Fabry, IOGS, CNRS, Université Paris Saclay, F91127 Palaiseau, France

## Abstract

We present a heuristic derivation of Born’s rule and unitary transforms in Quantum Mechanics, from a simple set of axioms built upon a physical phenomenology of quantization. This approach naturally leads to the usual quantum formalism, within a new realistic conceptual framework that is discussed in details. Physically, the structure of Quantum Mechanics appears as a result of the interplay between the quantized number of “modalities” accessible to a quantum system, and the continuum of “contexts” that are required to define these modalities. Mathematically, the Hilbert space structure appears as a consequence of a specific “extra-contextuality” of modalities, closely related to the hypothesis of Gleason’s theorem, and consistent with its conclusions.

A series of recent experimental tests of Bell’s theorem^1^,^2^,^3^ have been said to close the door on Einstein’s and Bohr quantum debate^4^. It is generally considered that Einstein lost the case, by advocating a notion of “local realism” incompatible with quantum mechanics (QM)^5^. However, Bohr^6^ also presented himself as a realist as far as physics is concerned, and QM has no direct conflict with relativistic causality. One may thus wonder whether a deep – but philosophically sound – redefinition of physical reality might provide a way to reconcile the founding fathers of quantum physics. In refs 7, 8, 9 and 10 we argued that this can be done, under the condition that fully predictable physical properties (called “elements of physical reality” in ref. 5) are attached not to a system alone, but to a system within a given experimental context^6^.

In this paper, we further exploit this idea, in order to present a heuristic derivation of the quantum formalism, understood as a non-classical way to calculate probabilities. An outstanding feature of our approach is that the superposition principle and Born’s rule appear as consequences of the quantized number of states accessible to a quantum system, without any appeal to “wave functions” – but we do recover projective measurements. Our approach bears some relationship with Gleason’s theorem^11^,^12^,^13^, as it will be discussed below (see also Methods).

We shall start without formalism, but from a few definitions and hypotheses, presented here as axioms. These axioms are based on standard quantum phenomenology, and they have been introduced and discussed in refs 7 and 8 under the acronym “CSM”, meaning Context, System, Modality. In the present article we will not repeat this discussion, but rather use the the following axioms to summarize the main features of our approach. Though the formulation of the axioms contains very little mathematics, they have deep mathematical consequences, that will be spelled out in the “Results” section below.

- **Axiom 1** (modalities): (i) Given a physical system, a *modality* is defined as the values of a complete set of physical quantities that can be predicted with certainty and measured repeatedly on this system. (ii) Here a “complete set” means the largest possible set compatible with certainty and repeatability, for all possible modalities attached to this set. This complete set of physical quantities is called a *context*, and the modality is attributed **jointly** to the system and the context. (iii) Modalities cannot show up independently of a context, but the same modality may appear in different contexts, with the same conditions of repeatability and certainty.
- **Axiom 2** (quantization): (i) For a given context, there exist *N* distinguishable modalities {*u*_*i*_}, that are mutually exclusive: if one modality is true, or realized, the others are wrong, or not realized. (ii) The value of *N*, called the dimension, is a characteristic property of a given quantum system, and is the same in all relevant contexts.
- **Axiom 3** (changing contexts): Given axioms 1 and 2, the different contexts relative to a given quantum system are related between themselves by continuous transformations *g* which are associative, have a neutral element (no change), and an inverse. Therefore the set of context transformations *g* has the structure of a continuous group 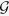.

For the sake of clarity, we note that, within the usual QM formalism (not used so far), a modality and a context correspond respectively to a pure quantum state, and to a complete set of commuting observables. The axioms are formulated for a finite *N*, but this restriction will be lifted below. Intuitively, as discussed in details in ref. 7, a context can be seen as a given “knob settings” of the measurement apparatus. We will not repeat this discussion here, but we want to consider the following question: it is postulated in Axiom 2 that there are *N* mutually exclusive modalities associated to each given context, but there are many more modalities, corresponding to all possible contexts, related according to Axiom 3. These modalities are generally not mutually exclusive, but are **incompatible**: it means that if one is true, one cannot tell whether the other one is true or wrong. Then, how to relate between themselves all these modalities?

A first crucial result already established in ref. 7 is that this connection can only be a probabilistic one, otherwise the axioms would be violated; the argument is as follows. Let us consider a single system, two different contexts *C*_*u*_ and *C*_*v*_, and the associated modalities *u*_*i*_ and *v*_*j*_, where *i* and *j* go from 1 to *N*. The quantization principle (Axiom 2) forbids to gather all the modalities *u*_*i*_ and *v*_*j*_ in a single set of more than *N* mutually exclusive modalities, since their number is fixed to *N*. Therefore the only relevant question to be answered by the theory is: If the initial modality is *u*_*i*_ in context *C*_*u*_, what is the *conditional probability* for obtaining modality *v*_*j*_ when the context is changed from *C*_*u*_ to *C*_*v*_? We emphasize that this probabilistic description is the unavoidable consequence of the impossibility to define a unique context making all modalities mutually exclusive, as it would be done in classical physics. It appears therefore as a joint consequence of the above Axioms 1 and 2, i.e. that modalities are quantized, and require a context to be defined.

Now, according to Axiom 3, changing the context results from changing the measurement apparatus at the macroscopic level, that is, “turning knobs”. A typical example is changing the orientations of a Stern-Gerlach magnet. These context transformations have the mathematical structure of a continuous group, denoted 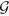: the combination of several transformations is associative and gives a new transformation, there is a neutral element (the identity), and each transformation has an inverse. Generally this group is not commutative: for instance, the three-dimensional rotations associated with the orientations of a Stern-Gerlach magnet do not commute. For a given context, there is a given set of *N* mutually exclusive modalities, denoted {*u*_*i*_}. By changing the context, one obtains *N* other mutually exclusive modalities, denoted {*v*_*j*_}, and one needs to build up a mathematical formalism, able to provide the probability that a given initial modality *u*_*i*_ ends up in a new modality *v*_*j*_.

The standard approach at this point is to postulate that each modality *u*_*i*_ is associated with a vector |*u*_*i*_〉 in a *N*-dimensional Hilbert space, and that the set of *N* mutually exclusive modalities in a given context is associated to a set of *N* orthonormal vectors. Rather than vectors |*u*_*i*_〉 and |*v*_*j*_〉, one can equivalently use rank-one projectors 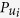 and 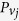, and Born’s rule giving the conditional probability *p(v*_*j*_|*u*_*i*_) can be written as


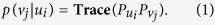


In this article, we will postulate neither Born’s rule nor even Hilbert spaces, but we will derive them as the consequence of the previous Axioms. Then we will discuss the relation with Gleason’s theorem, as well as some consequences of our approach.

## Results

In this part, we start from Axioms 1–3 and construct a consistent probability theory, by imposing some requirements on what it should describe. The first steps will thus be to translate the Axioms into mathematical constraints on probabilities relating modalities. This will lead us to manipulate *N* × *N* probability matrices, in a general way not restricted to the quantum formalism. Using our Axioms to obtain physically-based constraints, we will finally get Born’s rule and unitary transforms.

### First consequence of the Axioms: the general probability matrix

Let us denote {*u*_*i*_} and {*v*_*j*_} the respective modalities of the initial and final context, and define the probability 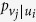 of finding the particular modality *v*_*j*_, when starting from modality *u*_*i*_. There are *N*^2^ such probabilities, that can be arranged in a *N* × *N* matrix 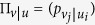, containing all probabilities connecting the *N* modalities in each context {*u*_*i*_} and {*v*_*j*_}. Since one has obviously 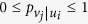 and 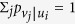, the matrix Π_*v*|*u*_ is said to be a *stochastic* matrix (see Methods for definitions).

For clarity, let us emphasize the interpretation of the conditional probability notation: in agreement with the definition of modalities as certainties, the meaning of 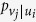 is that “if we start (with certainty) from modality *u*_*i*_ in the old context, then the probability to get modality *v*_*j*_ in the new context is 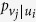
”. These probabilities provide the connection between theoretical predictions and experiments, and correspond to relative frequencies in repeated experiments starting from the same *u*_*i*_. It is not critical whether they are interpreted in a frequentist or Bayesian sense, but it is critical to acknowledge that they are intrinsic consequences of our axioms on quantized modalities, and thus are **not** associated to any “missing information”.

For *N* = 3, one has for instance


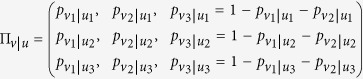


As we will see below, *N* ≥ 3 is required because some crucial properties of Π_*v*|*u*_ do not show up for *N* = 2. Let us also define a “return” probability matrix Π_*u*|*v*_, by exchanging the roles of the initial and final contexts. The matrix Π_*u*|*v*_ is stochastic like Π_*v*|*u*_, but these two matrices are *a priori* unrelated, whereas it is known that in standard QM, they are transpose of each other.

### Second consequence of the Axioms: the extra-contextuality of modalities

An essential ingredient for determining the mathematical structure of Π_*v*|*u*_ is provided by a physical constraint on the probability 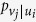. This is found in Axiom 1 (iii) where it was stated that “Modalities cannot show up independently of a context, but the same modality may appear in different contexts, with the same conditions of repeatability and certainty”. This means in particular that if 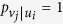, then *v*_*j*_ ≡ *u*_*i*_, i.e. *v*_*j*_ and *u*_*i*_ represent the same modality, within two different contexts. This claim may seem surprising since the measured physical quantities in the two contexts can be quite different (see example in Methods); but what matters here is that the certainty and reproducibility are transmitted from one context to the other, hence the idea that the modality is conserved. This has also a major mathematical consequence, which is that when 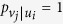, we will require that the **same mathematical object** is associated with the modality (*v*_*j*_ or *u*_*i*_) in the two contexts.

More generally, and again in agreement with the physical reality of modalities, we will require that the probability 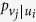 depends only on the particular modalities *u*_*i*_ and *v*_*j*_ being considered, and not on the whole contexts in which they are embedded. Importantly, to build our formalism, we shall apply this requirement not only to the value of 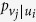, but also to its mathematical expression; how to do that will be spelled out below. This property will be called **“extra-contextuality”** (see relation with other works in Methods), and it means also that a modality can be defined independently of the (*N* − 1) other modalities which appear in a given context. Such an extra-contextuality is fully compatible with contextual objectivity^7^,^9^,^10^: the latter states that a modality needs a context to be defined, whereas the former tells that the same modality can show up in several contexts (a simple example is given in Methods, as well as an interesting link with a proof by John Bell^14^).

### The mathematical translation of the Axioms

Summarizing the previous discussions, we want to calculate probabilities relating physical events called modalities, occurring for a given physical system in a given physical context. Given the physical system, the rules are:

- **For any given physical context, there are exactly** *N* **mutually exclusive modalities.** As a consequence, the *N*^2^ probabilities 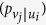 connecting the *N* modalities {*u*_*i*_} (resp. {*v*_*j*_}) in two different contexts can be arranged in a *N* × *N* stochastic matrix Π_*v*|*u*_. A similar “return” probability matrix Π_*u*|*v*_ is defined by exchanging the roles of the initial and final contexts, and the set of context transformations has the structure of a continuous group.
- **If** 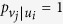
**, then** *v*_*j*_ **and** *u*_*i*_ **are the same modality, and will be associated with the same mathematical object.** This rule applies within a given context, where one has 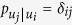. If all probabilities 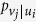 are either zero or one between two different contexts, one will say that this is the same context, up to re-labelling the modalities.
- **Extra-contextuality constraint (ECC): the probability** 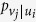 **depends only on the two modalities** *u*_*i*_ **and** *v*_*j*_ **being considered, and not on the whole contexts in which they are embedded. In mathematical terms,** 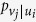 **depends only on the two mathematical objects associated with the two modalities.**

These rules have obtained from the Axioms, though not by a fully formal deduction. They may thus be considered as additional principles, deduced from the non-mathematical Axioms, and leading to exploitable mathematical consequences.

From there, the main idea of our derivation is the following: we will first write a general parametrization of stochastic matrices, which is mathematically and physically neutral, i.e., it is just a rewriting. Nevertheless, this parametrization provides a simple criterion for the stochastic matrix to be *unistochastic*, i.e. that its coefficients are the square moduli of the coefficients of a unitary matrix (see definitions in Methods). Then we will translate the extra-contextuality constraint into an equation, from which we will show that the matrices Π_*v*|*u*_ and Π_*u*|*v*_ are unistochastic. Finally the usual formalism of quantum mechanics (Born’s rule, unitary transforms, link between Π_*v*|*u*_ and Π_*u*|*v*_) will follow automatically.

### Mathematical lemmas on stochastic matrices

The theorems below are valid both for Π_*u*|*v*_ and Π_*v*|*u*_, so *u* and *v* will be omitted whenever clarity allows.

**Lemma 1:** The elements *p*_*j*|*i*_ of a *N* × *N* stochastic matrix can always be written under the general form


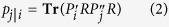


where 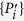 and 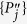 are two sets of *N* hermitian projectors of dimension *N* × *N*, mutually orthogonal within each set, and where *R* is a real nonnegative diagonal matrix such that **Tr**(*R*^2^) = *N*, and **Tr**(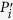 *R*^2^) = 1 for all projectors 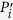 within 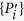.

*Proof.* Let us first introduce the orthogonal (*N* × *N*) projectors *P*_*i*_, that are zero everywhere, except for the *i*^*th*^ term on the diagonal that is equal to 1; one has obviously *P*_*i*_*P*_*j*_ = *P*_*i*_*δ*_*ij*_. A useful operation is then to extract the particular probability 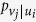 from Π_*v*|*u*_, or 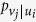 from Π_*u*|*v*_, and one has the following identities:


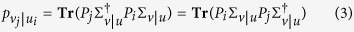



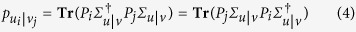


where **Tr** is the Trace, ^†^ is the Hermitian conjugate, and


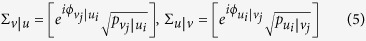


are *N* × *N* matrices formed by square roots of the probabilities, and by arbitrary phase factors which are introduced here for the sake of generality, and cancel out when calculating the matrices Π_*v*|*u*_ and Π_*u*|*v*_.

From Eqs (3,4,5) the elements *p*_*j*|*i*_ of a general stochastic matrix Π can be written as (the subcripts *u*|*v* or *v*|*u* are omitted for simplicity):


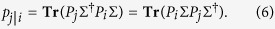


Now, according to the singular values theorem (see Methods), there must exist two unitary matrices *U* and *V*, and a real diagonal matrix *R*, such that


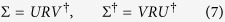


where the diagonal values of *R* are the square roots of the (real) eigenvalues of ΣΣ^†^, equal to those of Σ^†^Σ, and are called the singular values of Σ (see proof in Methods). The value of **Tr**(*R*^2^) is the sum of the square moduli of all the coefficients of Σ, and is therefore equal to *N*.

Using Eqs (6 and 7) *p*_*j*|*i*_ can now be written as:


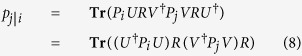



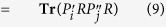


where {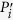 = *U*^†^*P*_*i*_*U*} and {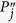 = *V*^†^*P*_*j*_*V*} are two sets of projectors, mutually orthogonal within each set. Finally, the normalization condition ∑_*j*_*p*_*j*|*i*_ = 1 implies that


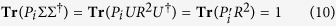


hence the diagonal elements of *R*^2^ in the basis associated with the projectors {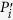} are all equal to one. ◻

Let us show now that very different situations occur, depending on the fact that the matrix *R* is (or is not) equal to the identity matrix 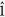. This is related to:

**Lemma 2:** The matrix Σ is unitary iff 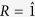.

*Proof.* Eq. (7) shows that Σ is unitary if 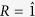, and if Σ is unitary then 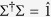 and 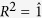, so 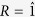. ◻

An important corollary is that the matrix Π is unistochastic if 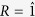. The reciprocal is not true, because Π being unistochastic does not imply that any matrix Σ defined by Eq. (5) is unitary (the phases may be wrong).

An obvious consequence of Lemma 2 is that if 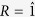 for all possible pairs of contexts, then the matrix Π is unistochastic for all pairs of context; we will show below that this corresponds to the usual quantum formalism. The opposite case is that 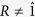 for some pairs of contexts, but we will show that this contradicts our basic constraint that 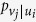 should depend only on the particular modalities *u*_*i*_ and *v*_*j*_ being considered. First let us establish the following mathematical Lemma:

**Lemma 3:** If 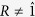 then 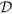 = 0, where 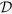 is the determinant of the unistochastic matrix obtained from the square moduli of the coefficients of *U* introduced in Lemma 1.

*Proof.* Let us consider the normalization conditions **Tr**(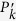 *R*^2^) = 1 obtained from Eq. (10), where the *N* projectors 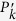 = *U*^†^*P*_*k*_*U* correspond to the initial context. Since one has also **Tr**(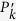) = 1, the *N* homogeneous equations 
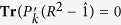
 must be verified by the *N* diagonal values of *R*^2^. This set of equations admits a non-trivial solution 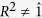 if its determinant 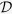 is equal to zero, and it is easy to check that 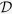 is the determinant of the unistochastic matrix obtained from *U*. ◻

Summarizing the previous results, Lemma 1 tells us that for any stochastic matrix Π, one can parametrize the probabilities *p*_*j*|*i*_ by using the diagonal matrix *R* and two sets of projectors 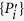 and 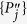. Then according to Lemmas 2 and 3, two situations are possible:

- either 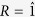 for all pairs of contexts, and the matrix Π is always unistochastic as shown in Lemma 2.

- or 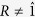 for some pairs of contexts, and a stochastic (but generally not unistochastic) matrix Π is obtained for appropriate projectors, with 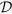 = 0 as shown in Lemma 3.

### The Fundamental Theorem

We are now in a position to use the extra-contextuality constraint (ECC), which says that the expression of *p*_*j*|*i*_ = **Tr**(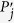 *R* 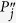 *R*) should depend only on the particular modalities *u*_*i*_ and *v*_*j*_ being considered, and not on the whole contexts in which they are embedded. A first step is the following Lemma:

**Lemma 4:** Given a N dimensional system, each context must be associated with a set of *N* mutually orthogonal projectors, each projector corresponding to one of the *N* mutually exclusive modalities.

*Proof.* In the case where the initial and final contexts are the same, then 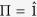, Σ is unitary and diagonal, and 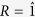. From its definition *V* can be any unitary matrix, and *U* = Σ*V*, so the two sets of projectors {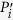} and {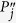} are identical, and are associated with the current context. In addition, since Lemma 2 gives *p*_*j*|*i*_ = **Tr**(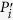

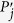
) = *δ*_*ij*_, each modality *u*_*k*_ (*k* = *i* or *j*) must be associated with a projector 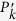 of the set {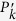} corresponding to the current context. ◻

For *N* ≥ 3, these *N* projectors may be part of other orthogonal sets, and the corresponding modalities may be part of other contexts. Again for consistency with the ECC, we will require that the same projector always corresponds to the same modality. This will extend first to all contexts containing one (or several) of the initial modalities, giving new projectors and new modalities, and then to the whole space of all *N* × *N* projectors, which will thus be associated to all possible modalities. This association has to be consistent when the contexts are changed; this will be discussed in eqs (14 and 15).

Let us emphasize that at this point we don’t have QM yet; in some sense, we have justified the Hilbert space framework of Gleason’s theorem, as the space of *N* × *N* projectors, but we still miss the main hypothesis and the result of the theorem, i.e. Born’s law. More precisely, we have justified that 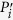 and 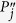 depend solely on *u*_*i*_ and *v*_*j*_ in eq. (2); however, it is still possible that *R* depends on the whole contexts *C*_*u*_ and *C*_*v*_ in which *u*_*i*_ and *v*_*j*_ are embedded, and not on these two modalities only.

So we will use again the ECC to require that not only 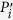 and 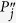 but also *R* depend solely on *u*_*i*_ and *v*_*j*_; more explicitly, this can be written:


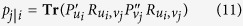


where 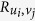 depends only on the two specific modalities *u*_*i*_, *v*_*j*_ associated with the projectors 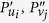, and not on the contexts in which they are embedded.

**Fundamental Theorem**: If each modality is bijectively associated with a rank-one projector, and if *p*_*j*|*i*_ is given by Eq. (11), then 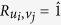 for all pairs of contexts.

*Proof.* Let us assume that 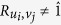; then one has 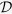 = 0 according to Lemma 3. But one can change the initial context by choosing new projectors 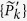 to replace the {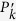}, keeping 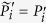 for the modality *i* of interest, whereas the other projectors are different, but still mutually orthogonal (this is possible only if *N* ≥ 3). Then 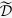 will generally not be zero any more (see Methods), so the assumption 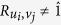 is not acceptable. More generally, the same reasoning is valid for any pair of modalities *u*_*i*_, *v*_*j*_ therefore one has 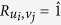 for all pairs of modalities, and also for all pairs of contexts. ◻

Intuitively, the theorem says that the resulting formula 
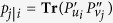
 provides the unique way to express the coefficients *p*_*j*|*i*_ of a stochastic matrix as a function of the sole modalities *u*_*i*_ and *v*_*j*_, satisfying the ECC as expressed by Eq. (11). As we will show now Born’s formula and unitary transforms directly follow from this result.

### Unitary matrices and Born’s formula

From now on we will take 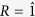 according to the previous Theorem. Therefore the matrix Σ_*v*|*u*_ = *UV*^†^ is unitary, but one may wonder whether orthogonal (real) matrices might be enough. In order to justify that the full unitary set is required, we will use Axiom 3, telling that the change of contexts corresponds to a continuous group, to require that the set of matrices Σ_*v*|*u*_ is connected in a topological sense, and contains the identity matrix. This set must contain permutation matrices, because they correspond simply to “relabelling” the modalities, i.e. to a trivial change of context. One has then

**Lemma 5:** If the set of matrices Σ_*v*|*u*_, including permutation matrices, is connected in a topological sense, and contains the identity matrix, then the matrices Σ_*v*|*u*_ must be complex unitary matrices.

*Proof.* The set of real orthogonal matrices is topologically disconnected in two parts with determinant +1 and −1, whereas permutation matrices may have determinant −1, and the identity has determinant +1. On the other hand, all (complex) unitary matrices are connected to the identity, hence the result (see also refs 15 and 16). ◻

We are thus lead to the conclusion that Σ_*v*|*u*_ must be a unitary matrix *S*_*v*|*u*_, with 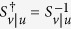. Then Eqs (3) for picking up a particular probability become:


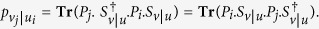


As said above Π_*v*|*u*_ is unistochastic, and we can define


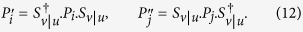


It is clear that these operators are all Hermitian projectors, i.e. one has *P*^†^ = *P* and *P*^2^ = *P* for each of them, and also that all sets {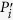} and {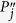} have the same orthogonality properties as the initial set of projectors {*P*_*i*_}, i.e. *P*_*i*_*P*_*j*_ = *P*_*i*_*δ*_*ij*_. One can thus rewrite Eq. (3) as:


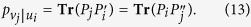


which is just Born’s formula (Eq. 1). Eqs (12 and 13) are consistent with our initial requirement associating a projector with a modality in any context, but make clear that this association is up to a global unitary transform, related to the choice of a fiducial context. In particular, there are two possible choices for the matrix Π_*v*|*u*_:


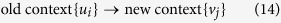



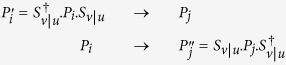


One can now come back to the matrix Π_*u*|*v*_, for which the same reasoning is valid, and leads to a unitary matrix *S*_*u*|*v*_. By reverting the contexts one has thus:


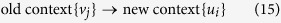



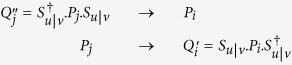


Again, the projectors should be the same for a given modality in a given context, i.e. one should have 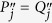 (for the same *P*_*i*_ in the other context), and 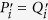 (for the same *P*_*j*_ in the other context). This is obtained if *S*_*u*|*v*_ is the inverse of *S*_*v*|*u*_, leading to a last lemma:

**Lemma 6:** If 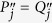 and 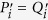 as defined above, then 
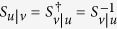
 (up to global phase factors), and the matrices Π_*u*|*v*_ and Π_*v*|*u*_ are related by 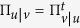.

*Proof*. Obvious from the relations (14) and (15). ◻

Then the various points of view represented in the relations (14, 15) are all consistent and give the same values for the probabilities, because each *S*_*v*|*u*_ can be associated to an element of the group of context transformations 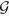, and its inverse is 
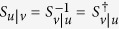
. For the general consistency of the approach including Axiom 3, this set of matrices gives a *N* × *N* (projective) representation of the group of context transformations; this is fully consistent with the well known Wigner theorem^17^. This continuous unitary evolution will be essential to describe the evolution of the system (translation in time)^17^. Since we have now reached the starting point of most QM textbooks^18^, it should be clear that the standard structure of QM can be obtained from this construction. In particular, one can associate the *N* orthogonal projectors {*P*_*i*_} to the *N* orthonormal vectors which are eigenstates of these projectors up to a phase factor, i.e., to rays in the Hilbert space. Similarly, the expected probability law for the measurement results {*a*_*i*_} will be obtained by writing any physical quantity *A* as an operator 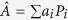, this is the usual spectral theorem. The tensor product structure for composite systems can also be introduced in the usual way (see Methods).

## Discussion

An interesting outcome of our derivation is that the usual Hilbert space structure (for *N* × *N* matrices) shows up, without any initial assumption of a superposition principle, interference effect, or wave function^19^,^20^,^21^,^22^,^23^,^24^,^25^,^26^. This structure comes directly from requirements on probabilities, implying that the matrices Π_*u*|*v*_ and Π_*v*|*u*_ belong to the unistochastic subset of stochastic matrices. This appears as the mathematical consequence of the joint physical requirements of contextuality of the theory (contexts are needed to define modalities), quantization of modalities (making probabilities necessary), and extra-contextuality of modalities (probabilities depend on modalities, that may belong to different contexts).

Extra-contextuality is also a crucial hypothesis for Gleason’s theorem^11^,^12^, which is deeply related to our derivation; however, the reasonings proceed in quite different ways. The Hilbert space structure is an assumption in Gleason’s theorem, whereas in our case it appears more heuristically. Rather than reconstructing “from scratch” the Trace formula, as done by Gleason, we introduce it as a general parametrization of stochastic matrices; this avoids the heavy machinery of Gleason’s theorem^11^,^12^, in particular the demonstration of continuity (see Methods). Then we use extra-contextuality to restrict acceptable matrices to unistochastic ones, ending up again with Born’s formula in finite dimension. Using explicitly Gleason’s theorem is also possible^13^, and has the advantage of lifting the restriction on a finite *N* (see Methods).

We emphasize that we do not need any additional “measurement postulate”, since measurement is already included in Axiom 1, i.e. in the very definition of a modality^7^,^8^,^9^,^10^. Quantum superposition are here as usual, but they are not spooky “dead-and-alive” concepts: they are rather the manifestation of a modality (i.e., a certainty) in another context. Entanglement is also present as linear superpositions of tensor product states, corresponding to modalities in a “joint” context, and the specific case of two-particle Bell-EPR experiment is discussed in ref. 8. Since a modality requires both a context and a system, it embeds non-local features corresponding to quantum non-locality, but it is fully compatible with relativistic causality^7^,^8^, and operationally agrees with no-signaling, just like QM does. From a foundational point of view, our approach also provides a clear distinction between the modality, which is a real physical phenomenon, or a physical event in the sense of probability theory, and the projector, which is a mathematical tool for calculating non-classical probabilities.

To conclude, let us emphasize that we discussed a very idealized version of QM, based on pure states and orthogonal measurements. Nevertheless, this idealized version does provide the basic quantum framework, and connects the experimental definition of a physical quantity and the measurement results in a consistent way, both physically and philosophically^7^. Adding more refined tools such as density matrices, imperfect measurements, POVM, open systems, decoherence, is of great practical interest and use, but this will not “soften” the basic ontology of the theory, as it is presented here. The present work, deeply rooted in ontology, is thus complementary to many recent related proposals^19^,^20^,^21^,^22^,^23^,^24^,^25^,^26^,^27^,^28^,^29^,^30^,^31^,^32^,^33^.

## Methods

### Stochastic matrices

A *stochastic* matrix has real positive coefficients, with all lines summing up to 1. *Bistochastic* matrices are stochastic ones, with both lines and columns summing to 1. *Orthostochastic* and *unistochastic* matrices are obtained by taking the square moduli of the coefficients of respectively an orthogonal or a unitary matrix^34^. For *N* = 2, all bistochastic matrices are also ortho- and uni-stochastic, and for *N* ≥ 3, the set of unistochastic matrices is larger than the orthostochastic set, but smaller than the bistochastic set. For instance, the simple matrix


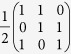


is a well known example of a bistochastic matrix, which is neither orthostochastic, nor unistochastic; therefore it is not an acceptable (quantum) probability matrix Π_*v*|*u*_.

### Singular values theorem and the invariance of R

To obtain the singular values decomposition, diagonalize the Hermitian matrix Σ^†^Σ, get the real diagonal matrix *R* and the unitary matrix *V* so that *V*^†^Σ^†^Σ*V* = *R*^2^. Then define another unitary matrix *U* such that *UR* = Σ*V*, and *RU*^†^ = *V*^†^Σ^†^, so that *U*^†^ΣΣ^†^*U* = *R*^2^. One gets thus the decomposition Σ = *URV*^†^ as expected.

In the demonstration of the fundamental theorem, we note that one might restrict the new projectors 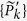 to be such that 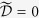. Then *R* can be different of 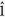, but it has still to be modified to some 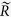 to fulfill the normalization conditions with the 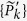. Since the hypotheses is that *R* should be constant, this case is excluded also.

One may also wonder what would happen if no phase factors were included in the definition of Σ. Then the Lemmas are still valid, but Σ cannot be unitary, and not even orthogonal. Then according to Lemma 2, *R* cannot be the identity, and therefore the extra-contextuality constraint cannot be satisfied.

### Extra-contextuality and Gleason’s theorem

Extra-contextuality is not a new concept, but it is a new name given to a known concept, called non-contextuality in articles dealing with Gleason’s theorem^12^, or “measurement non-contextuality” in ref. 31. Extra-contextuality is **not** the contrary of contextuality, and it avoids confusion arising when using “non-contextuality”. In particular, contexts are needed to define modalities, and modalities are extra-contextual, without any contradiction with refs 7, 9, 10 and 13 or with the Kochen-Specker theorem. As a simple example of extra-contextuality, consider a system of two spin 1/2 particles, and define 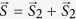. Using standard notations for coupled and uncoupled basis, the |*m*_1_ = 1/2, *m*_2_ = 1/2〉 modality in the context {*S*_*z*1_, *S*_*z*2_} is the same as the |*S* = 1, *m*_*S*_ = 1〉 modality in the context 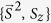, though other modalities in the same two contexts are different.

Demonstrating continuity of the probability formula is an essential step of Gleason’s theorem. In our derivation continuity appears formally in Axiom 3, and is embedded in the matrix formalism that we are using. It is used in the Fundamental theorem to to build a new set of projectors 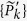, keeping one of them constant, and in Lemma 5 to get complex unitary matrices. So it does play a role, but does not have to be demonstrated. The explicit use of Gleason’s theorem for allowing the dimension *N* to be infinite is spelled out in ref. 13. This requires to introduce an Axiom 4 associating modalities and projectors in an Hilbert space; such an Axiom is not formally required in the present heuristic derivation, but it provides a useful “back-up”.

It is interesting to note that John Bell demonstrated explicitly in ref. 14 (Section V) that if extra-contextuality is accepted as it is done in the hypothesis of Gleason’s theorem, then the impossibility of hidden variables (HV) automatically follows (more technically, Bell showed that there is no dispersion-free state). Then he wrote at the end of his proof : *It was tacitly assumed that measurement of an observable must yield the same value independently of what other (commuting) measurements may be made simultaneously. Thus as well as P*(Φ_3_) *say (projector on vector* Φ_3_*), one might measure either P*(Φ_2_) *or P*(Ψ_2_)*, where* Φ_2_ *and* Ψ_2_ *are orthogonal to* Φ_3_ *but not to one another. These different possibilities require different experiment arrangements; there is no a priori reason to believe that the results for P*(Φ_3_) *should be the same.* So Bell rejected the “tacit assumption”, and therefore also the conclusion that there is no dispersion-free states. But extra-contextuality, seen as a consequence of the reality of modalities, may provide the missing “a priori reason” to accept the assumption, and thus also Bell’s proof.

Obviously Bell’s goal was very different from ours, since he was investigating the possibility of HV, whereas we want to recover the quantum formalism. Nevertheless, we conclude that if we accept extra-contextuality, then the quantum formalism follows (from the present article), and consistently with this result, HV are excluded (from ref. 14).

### Relations with textbook quantum mechanics

In this section we outline various issues relating our approach to standard QM. First, we considered only pure states and orthogonal (projective) measurements. This fits with the usual view that mixed states (density matrices) and non-orthogonal measurements (POVM) correspond to more classical aspects of probabilities, and can be introduced at a later stage. This is possible because in each context, a classical probability distribution can be built upon the *N* mutually exclusive modalities.

In our approach entanglement appears naturally in the following way: let us consider two systems 1 and 2 with *N*_1_ and *N*_2_ mutually exclusive modalities. If both systems are considered together, but each one in its own context, there are clearly *N* = *N*_1_ × *N*_2_ mutually exclusive modalities. But from Axiom 2, the value of *N* does not depend on the context, so the global system must be described by *N* × *N* projectors and unitary matrices. Many of these projectors cannot be split into projectors acting separately on system 1 or system 2, and are associated to entangled states.

In this article the postulate on time evolution is not spelled out, but it enters in the same framework, by including translations in time in the group 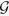. For instance, if 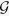 is the Galileo group, standard non-relativistic QM can be recovered, including Schrödinger’s equation^17^. Also, we did not discuss the known connection between the physical quantities and the infinitesimal generators of 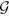, or the role of “projective” representations. Finally, we considered only non-relativistic quantum mechanics, and therefore “type I” von Neumann algebra, see e.g. ref. 35.

## Additional Information

**How to cite this article**: Auffèves, A. and Grangier, P. Recovering the quantum formalism from physically realist axioms. *Sci. Rep.* **7**, 43365; doi: 10.1038/srep43365 (2017).

**Publisher's note:** Springer Nature remains neutral with regard to jurisdictional claims in published maps and institutional affiliations.

## References

[b1] Hensen B. . “Loophole-free Bell Inequality Violation Using Electron Spins Separated by 1.3 Kilometres”. Nature 526, 682 (2015). 26503041 10.1038/nature15759

[b2] Giustina M. . “Significant-Loophole-Free Test of Bell’s Theorem with Entangled Photons”. Phys. Rev. Lett. 115, 250401 (2015). 26722905 10.1103/PhysRevLett.115.250401

[b3] Shalm L. K. . “Strong Loophole-Free Test of Local Realism”. Phys. Rev. Lett. 115, 250402 (2015). 26722906 10.1103/PhysRevLett.115.250402 PMC5815856

[b4] Aspect A. “Closing the Door on Einstein and Bohr’s Quantum Debate”. Physics 8, 123 (2015).

[b5] Einstein A., Podolsky B. & Rosen N. “Can Quantum- Mechanical Description of Physical Reality Be Considered Complete?”. Phys. Rev. 47, 777 (1935).

[b6] Bohr N. “Can Quantum-Mechanical Description of Physical Reality be Considered Complete?”. Phys. Rev. 48, 696 (1935).

[b7] Auffèves A. & Grangier P. “Contexts, Systems and Modalities: a new ontology for quantum mechanics”. Found. Phys. 46, 121 (2016), eprint arXiv:1409.2120 [quant-ph] (2014).

[b8] Auffèves A. & Grangier P. “Violation of Bell’s inequalities in a quantum realistic framework”. Int. J. Quantum Inform. 14, 1640002 (2016); eprint arXiv:1601.03966 [quant-ph] (2016).

[b9] Grangier P. “Contextual objectivity: a realistic interpretation of quantum mechanics”. European Journal of Physics 23(3), 331 (2002); eprint arXiv:quant-ph/0012122 (2000).

[b10] Grangier P. “Contextual objectivity and the quantum formalism”. International Journal of Quantum Information 3(1), 17–22 (2005); eprint arXiv:quant-ph/0407025 (2004).

[b11] Gleason A. M. “Measures on the Closed Subspaces of a Hilbert Space”. J. Math. Mech. 6(6), 885–893 (1957).

[b12] Granström H. *“Gleason’s theorem”*. Master Thesis, Department of Physics, Stockholm University (2006); http://kof.physto.se/theses/helena-master.pdf.

[b13] Auffèves A. & Grangier P. “*A simple derivation of Born’s rule with and without Gleason’s theorem*”. eprint arXiv:1505.01369 [quant-ph] (2015).

[b14] Bell J. S. “On the Problem of Hidden Variables in Quantum Mechanics”. Rev. Mod. Phys. 38, 447 (1966).

[b15] David F. “*A short introduction to the quantum formalism*[*s*]”. Lectures notes for a course given at IPhT-Saclay and Ecole Doctorale ED107 de Physique de la Region Parisienne, eprint arXiv:1211.5627 [math-ph] (2012).

[b16] Aaronson S. *“Is quantum mechanics an island in theory space”*. eprint arXiv:quant-ph/0401062 (2004).

[b17] Laloë F. *“Les symétries en mécanique quantique”*. Cours de DEA, Ecole Normale Supérieure (1980).

[b18] Cohen-Tannoudji C. Diu B. & Laloe F. *“Mécanique Quantique”*. Hermann, 1977.

[b19] Hardy L. “Why is nature described by quantum theory?” In “Science and Ultimate Reality”. pages 45–71, Cambridge University Press, 2004.

[b20] Hardy L. *Reformulating and Reconstructing Quantum Theory*. eprint arXiv:1104.2066v1 (2011).

[b21] Popescu S. & Rohrlich D. “Causality and Nonlocality as Axioms for Quantum Mechanics”. Proceedings of the Symposium on Causality and Locality in Modern Physics and Astronomy (York University, Toronto, 1997).

[b22] Brukner C. & Zeilinger A. “Information Invariance and Quantum Probabilities”. Found. Phys. 39, 677 (2009).

[b23] Pawlowski M. . “A new physical principle: Information Causality”. Nature 461, 1101 (2009). 19847260 10.1038/nature08400

[b24] Chiribella G., D’Ariano G. M. & Perinotti P. “Informational derivation of quantum theory”. Phys. Rev. A 84, 012311 (2011).

[b25] Colbeck R. & Renner R. “No extension of quantum theory can have improved predictive power”. Nature Commun. 2, 411 (2011). 21811240 10.1038/ncomms1416 PMC3265370

[b26] Masanes L., Müller M. P., Augusiak R. & Perez-Garcia D. “Existence of an information unit as a postulate of quantum theory”. PNAS 110:41, 16373 (2013). 24062431 10.1073/pnas.1304884110 PMC3799387

[b27] Zurek W. H. “Quantum darwinism, classical reality, and the randomness of quantum jumps”. Phys. Today 67, 44–50 (October 2014).

[b28] Auffèves A., Grangier P., Kastner R. E. & Zurek H. W. “Classical selection and quantum Darwinism”. Phys. Today 68, 8–10 (May 2015).

[b29] Amaral B., Terra Cunha M. & Cabello A. “The exclusivity principle forbids sets of correlations larger than the quantum set”. Phys. Rev. A 89, 030101 (2014).

[b30] Sainz A. B. . “Exploring the Local Orthogonality Principle”. Phys. Rev. A 89, 032117 (2014).

[b31] Acin A., Fritz T., Leverrier A. & Sainz A. B. “A Combinatorial Approach to Nonlocality and Contextuality”. Commun. Math. Phys. 334(2), 533 (2015).

[b32] Ekert A. “Quantum Paths” (eds Hui Khoon N. g. & Rui Han), 199–203 (World Scientific, 2015).

[b33] Horodecki R., Korbicz K. & Horodecki P. “Quantum origins of objectivity”. Phys. Rev. A 91, 032122 (2015).

[b34] Bengtsson I., Ericsson A., Ku s M., Tadej W. & Zyczkowski K. “Birkhoff’s polytope and unistochastic matrices, *N* = *3* and *N* = *4*”. Commun. Math. Phys. 259, 307–324 (2005); eprint arXiv:math/0402325 (2004).

[b35] Rèdei M. & Summers S. J. “Quantum probability theory”. Studies in the History and Philosophy of Modern Physics 38, 390–417 (2007); eprint arxiv:quant-ph/0601158 (2006).

